# Malignant Peripheral Nerve Sheath Tumor of the Hand: A Case Report

**DOI:** 10.7759/cureus.39205

**Published:** 2023-05-18

**Authors:** Sangita Jogdand, Rahul Rajendran

**Affiliations:** 1 Department of Surgery, Jawaharlal Nehru Medical College, Datta Meghe Institute of Medical Sciences, Wardha, IND

**Keywords:** malignant peripheral nerve sheath tumor, solitary neurofibroma, hand tumor, radiotherapy, sarcoma

## Abstract

An uncommon and aggressive type of soft tissue sarcoma that develops from peripheral nerves is known as a malignant peripheral nerve sheath tumor (MPNST). It is typically associated with neurofibromatosis type 1. Hence, this case report presents a case of a 42-year-old woman with a mass over the palmar aspect of her right hand that had been slowly growing over the previous year. Complete resection of the tumor with groin flap was performed followed by adjuvant radiotherapy. Over the last year, the patient has been monitored on an outpatient basis without displaying any signs of a local recurrence.

## Introduction

Schwann cell-derived malignant peripheral nerve sheath tumors (MPNST) typically affect peripheral nerves or the nerve sheath. They are extremely rare to arise over the palm of the hand and usually have a gradual progression followed by a sudden rapid increase in growth [1]. These tumors are mostly painless but can produce compressive symptoms such as motor and sensory dysfunction [2]. MPNST are extremely aggressive tumors and can be locally invasive. These tumors also tend to have lymphatic and hematogenous spread along with a high recurrence rate [3]. The sensitive markers involved consist of S-100 and SOX10 [4]. The diagnosis of MPNST in a patient without diagnosed neurofibromatosis needs to be evaluated carefully on the basis of the presence of the spindle cell sarcoma markers [5].

## Case presentation

**Figure 1:**
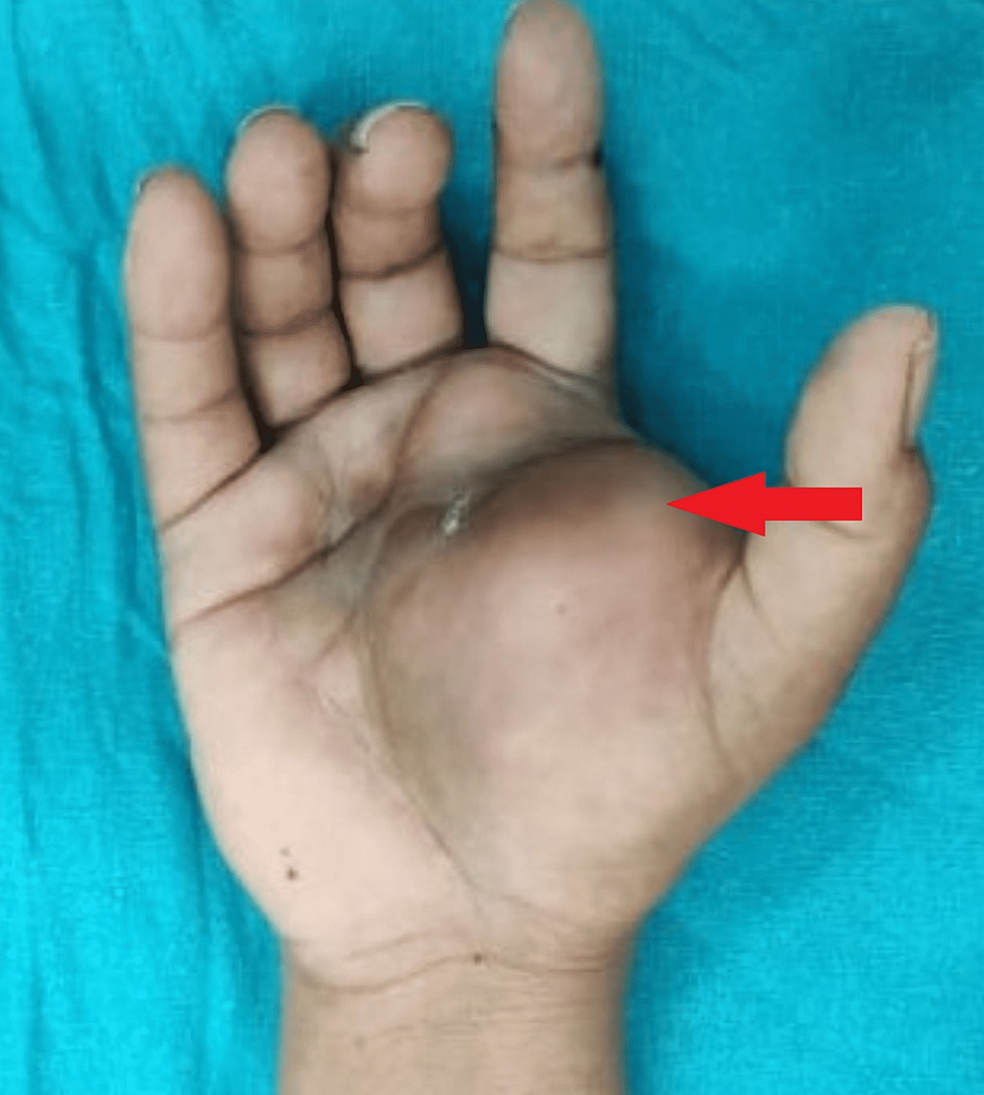
Clinical image of swelling over right hand.

A 42-year-old female reported with complaints of right palm edema that had been present for a year. The swelling progressively increased in size over the year and was associated with pain for the last couple of months. History of excision attempted three months back was present. Local examination revealed a globular, firm, non-tender, non-compressable, non-mobile swelling over the thenar eminence of the right palm. The overlying skin was however normal (Figure 1). 

**Figure 2:**
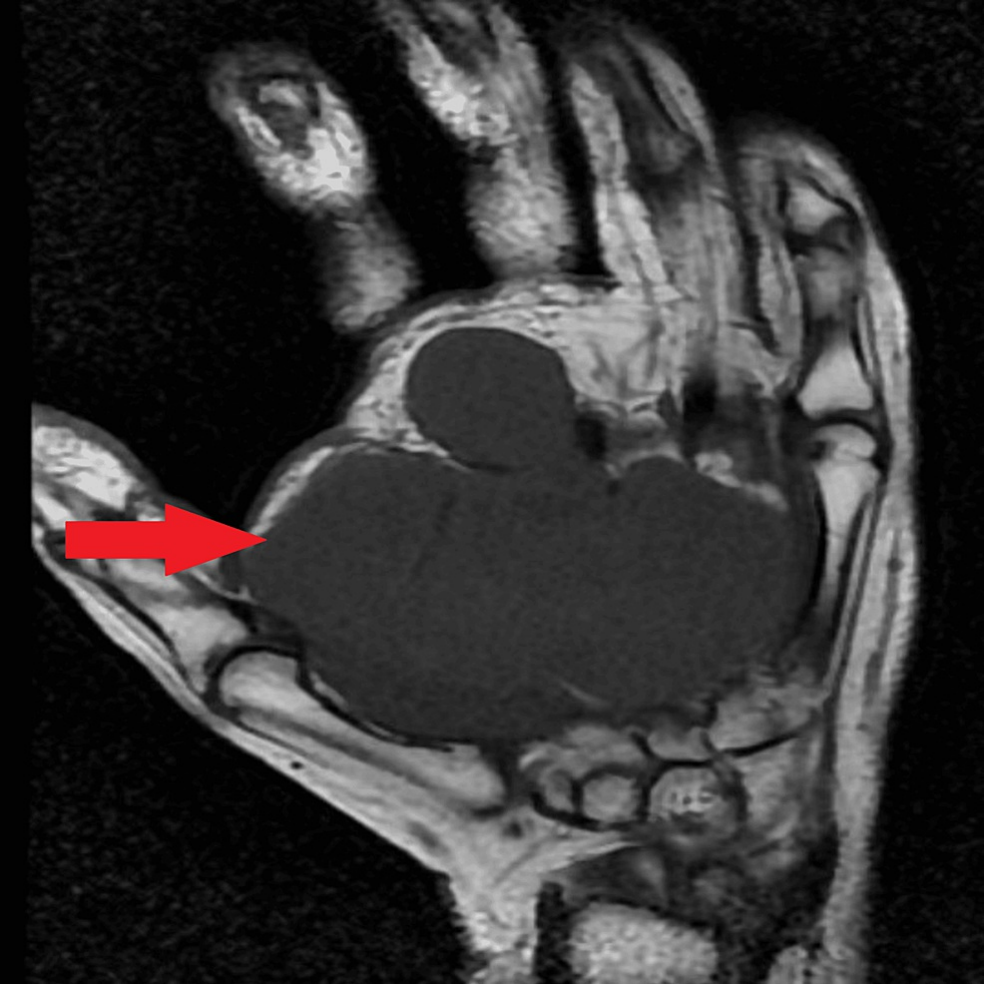
T1 weighted image of MRI MRI= Magnetic resonance imaging

**Figure 3:**
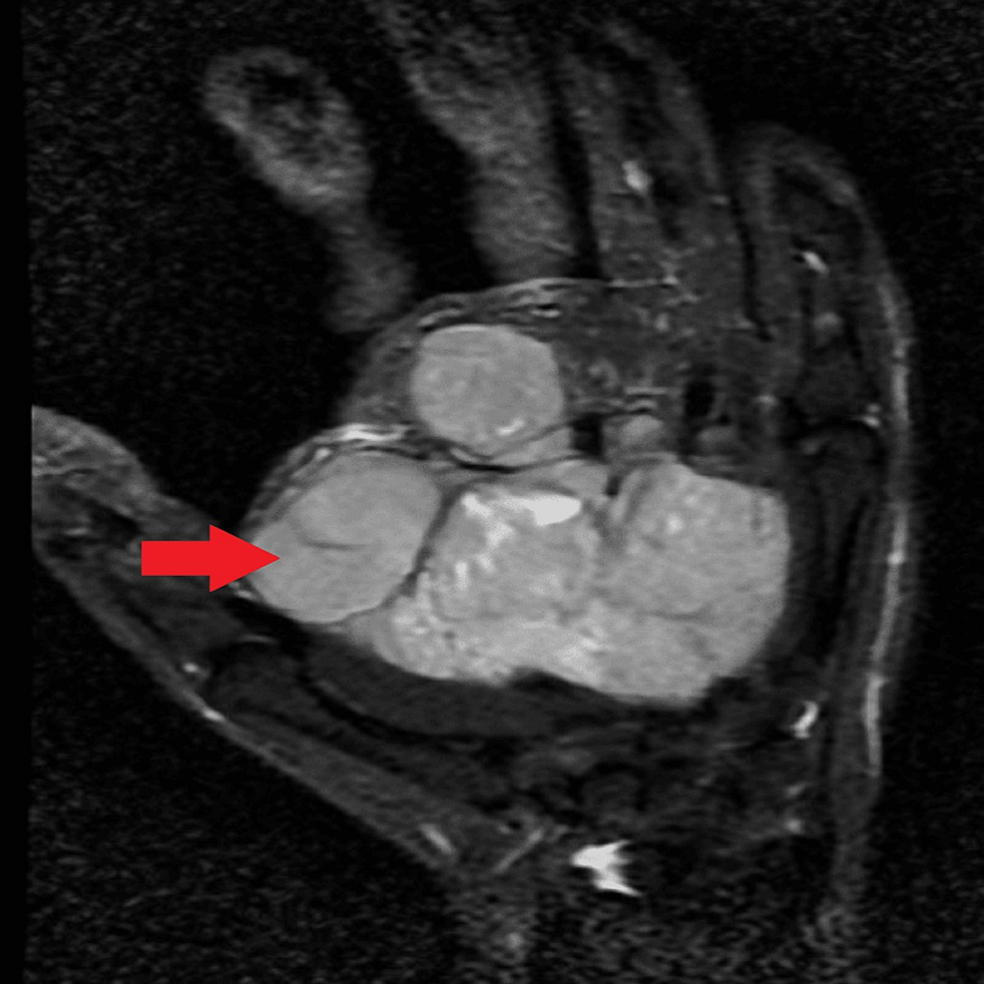
T2 weighted image of MRI MRI= Magnetic resonance imaging

Ultrasound of the subcutaneous plane of the palmar aspect on the right hand showed evidence of a well-defined large multilobulated lesion of size approximately 7.0 x 7.1 x 3.4 cm heterogeneously hypoechoic in the echotexture with high vascularity on color Doppler study. The Magnetic Resonance Imaging (MRI) showed a well-defined multilobulated lesion 7.3 cm x 7.1 cm x 3.5 cm in size over the right palm. On T1-weighted imaging (Figure 2), it was uniformly hyperintense to muscles and homogeneously hypointense to muscles on T2-weighted/short-tau inversion recovery (STIR) images (Figure 3), producing encasement of the forearm's flexor tendons and involvement of the median nerve at the proximal aspect. 

**Figure 4:**
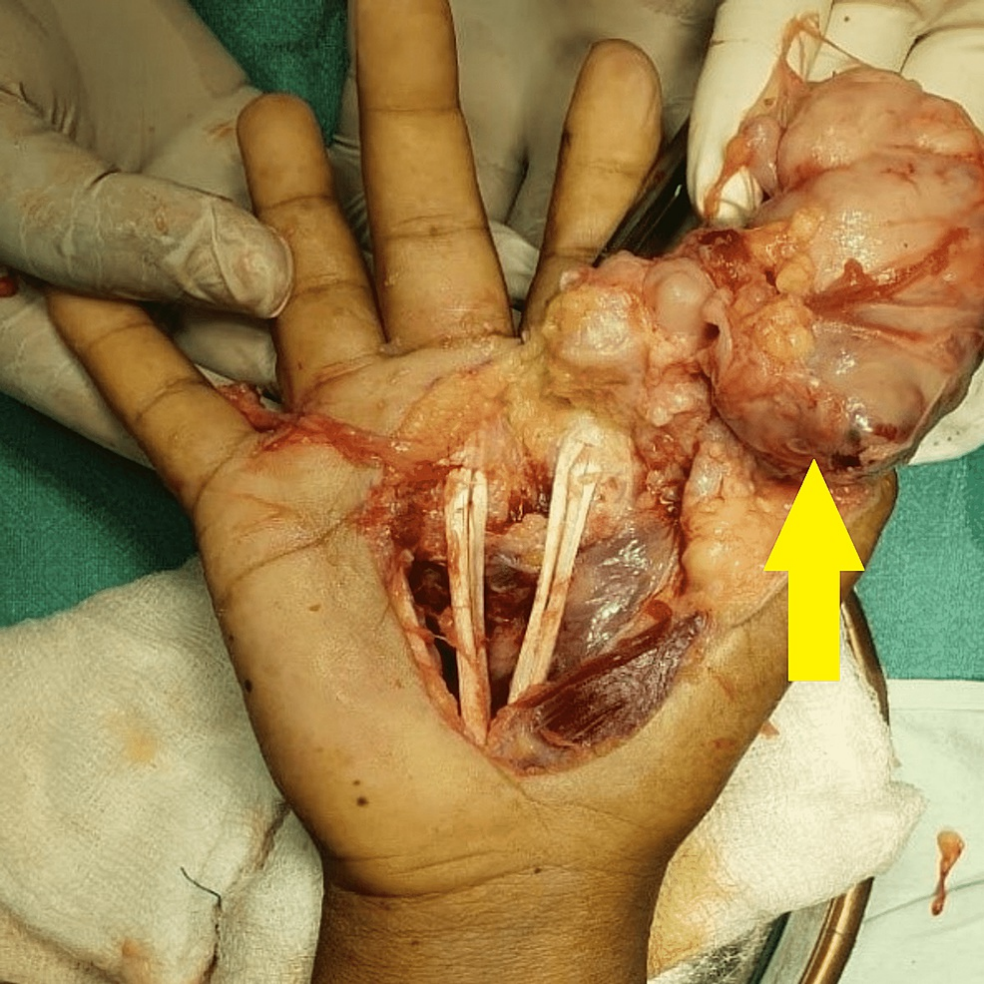
Intraoperative image of tumor.

In the hemorrhagic background, fine needle aspiration cytology (FNAC) revealed minimal cellularity, some neural cells, and scattered cells with modest pleomorphism. Complete surgical excision of the mass (Figure 4) with groin flap followed by flap release was carried out. 

**Figure 5:**
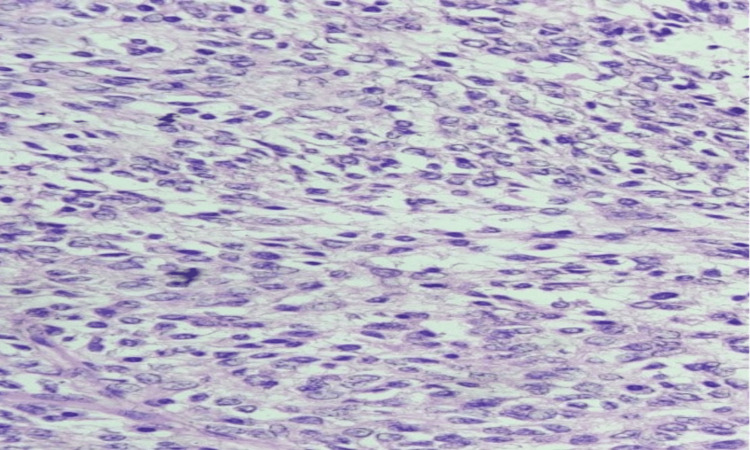
40x Image showing monomorphic spindle cells arranged in interlacing fascicles, palisades, and whorls

However, sections from the medial, lateral, superior, and inferior borders were free from infiltration by cancerous cells. The histopathological analysis of the excised specimen revealed a 40x image showing monomorphic spindle cells arranged in interlacing fascicles, palisades, and whorls (Figure 5) which characteristically indicated MPNST (low grade), and Fédération Nationale des Centres de Lutte Contre le Cancer (FNCLCC) Grade 1 (score 3), with lymphovascular invasion, capsular invasion, and tumor necrosis present. The presence of S-100 and neuron-specific enolase (NSE) markers ruled out other spindle cell sarcomas. The patient underwent adjuvant radiotherapy as per the tumor board's advice and has been under follow-up for one year with no evidence of recurrence. 

## Discussion

MPNST are extremely rare nervous system tumors that arise from fibroblast or Schwann cells of peripheral nerve sheaths [6,7]. MPNST belongs to the subcategory of soft tissue sarcomas as per the World Health Organization (WHO) central nervous system tumors classification [8]. Malignant sarcomas are relatively uncommon, making up just 1% to 2% of hand tumors. In patients with neurofibromatosis type 1 (NF1), the annual incidence of MPNST is between 2 and 5%, compared to 0.001% in the general population [9]. MPNST can develop over peripheral nerves in any part of the body theoretically but is commonly found to involve large nerves of the trunk and proximal limbs such as the sciatic nerve and brachial plexus.

Most cases are clinically present as rapidly enlarging soft-tissue masses with neuropathic symptoms such as paresthesias, radicular pain, or motor deficits. It is difficult to differentiate MPNST from benign tumors as there is both radiological and histological resemblance. According to Demir et al. [10], the clinical diagnosis of MPNST is simple in patients who do not have NF1 and who present with a palpable mass and pain. In contrast, patients who do have NF1 may experience a delay in diagnosis because they may be misdiagnosed as having classical neurofibroma or plexiform neurofibroma, which can cause further delay [10]. In cases of suspected malignancy, MRI and biopsy should be carried out [11,12]. Since T1-weighted pictures exhibit low to intermediate signal intensity whereas, T2-weighted sequences show high signal intensity with heterogeneous enhancement and plexiform neurofibromas; this demonstrates that MPNSTs have several MRI characteristics in common. According to Wasa et al. [13], individuals with MPNST show intratumoral cystic regions, enormous masses, peripheral enhancement patterns, and edema-like zones [13]. According to histological descriptions, spindle cells in MPNSTs exhibit hyperchromaticity and have a fasciculated pattern. Other prevalent findings include perivascular hypercellularity, regions of necrosis, elongated and wavy nuclei, and high mitotic activity [14].

Due to their histologic similarity, spindle cell sarcomas must be distinguished from one another using immunologic markers and sufficient pathologic samples [5]. The markers are S-100 and NSE for tumors of the MPNST type, smooth muscle actin (SMA), desmin, and vimentin for leiomyosarcoma, S-100, epithelial membrane antigen (EMA), and vimentin for Schwannoma, cytokeratin and vimentin for synovial sarcoma, vimentin for fibrosarcoma, and S-100, melanin, and glycogen for clear cell sarcoma [14]. Cellular schwannoma, dermatofibrosarcoma protuberans, melanoma, leiomyosarcoma, MPNST, synovial sarcoma, and clear cell sarcoma are among the differential diagnoses for soft-tissue spindle cell sarcomas [15]. The lungs, liver, and bones are the most typical sites for local recurrence and metastases in MPNST, which are regarded as high-grade tumors. Wide local excision with 3 cm margins is the primary treatment option for soft-tissue sarcomas. But when R0 resection is not possible or if the tumor is of high grade, large-size surgery is followed by adjuvant radiation therapy to minimize the chance of local recurrence. Chemotherapy has shown a limited role in spindle cell sarcomas. MPNST poses a significant diagnostic difficulty for both surgeons and pathologists. This case brings to light the necessity of being vigilant and aggressive management of a potentially malignant mass.

## Conclusions

It is quite challenging to identify and manage soft tissue sarcomas occurring over uncommon parts of the body. This case enhances understanding on how to approach and treat similar cases in the future. It also justifies that aggressive management in such cases improves both functional and survival outcome of the patient.
